# Coexistence of Relapsing Polychondritis and Sickle Cell Disease in a Child

**DOI:** 10.1155/2021/3600451

**Published:** 2021-11-24

**Authors:** Bernard Ofoe Tetteh, Florence-Barbara Yebuah, Maame-Boatemaa Amissah-Arthur, Dzifa Dey

**Affiliations:** ^1^Rheumatology Unit, Department of Internal Medicine and Therapeutics, Korle-Bu Teaching Hospital, Accra, Ghana; ^2^Ophthalmology Department, Korle-Bu Teaching Hospital, Accra, Ghana; ^3^Rheumatology Unit, Department of Internal Medicine and Therapeutics, University of Ghana Medical School, Accra, Ghana

## Abstract

Relapsing polychondritis (RP) is a rare, severe connective tissue disease of unknown etiology affecting cartilaginous and proteoglycan-rich structures in an episodic and inflammatory manner. Approximately a third of RP cases occur in conjunction with another disease usually systemic autoimmune rheumatic disease, or myelodysplastic syndrome. Sickle cell disease (SCD) is a common inherited hematologic condition characterized by the inheritance of two abnormal hemoglobins, of which one is a hemoglobin S, presenting with severe acute and chronic complications from vaso-occlusive phenomena, which can be difficult to differentiate from RP. The pathogenesis of RP is poorly understood but suggests an autoimmune mechanism with a link to sickle cell disease yet to be established. Treatment is empiric with steroids, anti-inflammatory, and disease-modifying antirheumatic drugs being the mainstay of therapy. Severe complications occur despite treatment, with respiratory involvement being the most catastrophic. This case report reviews a complex case of RP in an 11-year-old girl with sickle cell disease (SF genotype) presenting with bilateral red painful eyes, a painful swollen left ear, and knee pain. Laboratory findings revealed elevated inflammatory markers with negative immune serology. A diagnosis of RP was made based on the patient's symptomatology, presentation, and fulfillment of 5 out of the 6 clinical features using McAdam's criteria. Management was instituted with a myriad of conventional and biologic DMARDs and other anti-inflammatory medications with no significant improvement and the development of complications of airway obstruction from disease activity and osteoporotic fracture from steroid therapy and underlying hemoglobinopathy. In children, the diagnosis of RP is delayed or overlooked due to its low incidence, variability in clinical symptoms, or sharing similar clinical features with other coexisting disease entities. This article reports its occurrence in the pediatric population and highlights the difficulty in managing such cases as there are no defined standard treatment protocols.

## 1. Introduction

Relapsing polychondritis (RP) is a rare, severe connective tissue disease with an episodic and progressive inflammatory nature involving cartilaginous structures, mainly the ear, nose, and laryngotracheobronchial tree. The disease also affects the joints, sclera, and various proteoglycan-rich tissues including the media of arteries [1].

Sickle cell disease (SCD) refers to a group of disorders of hemoglobin in which the sickle mutation is coinherited with a mutation at the other beta globin allele that reduces normal beta globin production. These include sickle cell anemia, sickle cell thalassemia, hemoglobin SC disease, and hemoglobin SF disease, which our patient had. SCD presents with severe acute and chronic complications involving many organs in the form of acute pain episodes, anemia, recurrent infections, and chronic end organ damage [2].

RP is common in patients aged 40–50 years but may occur at any age with a genetic link suggested by a significantly higher frequency of HLA-DR4 in patients with RP than in healthy individuals [1] and shows various immune responses directed against type 2 collagen and matrilin-1 [3].

Approximately 30% of RP cases coexist with other autoimmune inflammatory disorders with a small percentage being associated with nonrheumatic conditions [4]. However, the coexistence of RP and SCD has not been reported, and the management of such cases remains uncertain.

RP is thought to be very rare among individuals of African descent with the majority of patients being of Caucasian origin [5]. Pediatric RP accounts for less than 5% of the cases reported, and it is characterized by frequent hospitalizations and emergency room visits, missed school attendance, and significant disability [6].

The most common clinical features of RP are chondritis and polyarthritis.

Bilateral auricular chondritis is the most common feature observed in 90% of cases during the course of the disease, and this presents as painful, red, violaceous edema confined to the cartilaginous part of the ear. This leads to deformation of the ear following repeated flares and resembles the cauliflower ear of professional boxers. They can also present with either conductive or sensorineural hearing loss, saddle nose deformity, and laryngotracheobronchial involvement. Nonerosive asymmetric arthritis, ocular manifestations such as episcleritis, scleritis, uveitis, retinopathy, and conjunctivitis are also features of the disease [7]. Less frequently, patients can present with neurological involvement in the form of headaches, vasculitis, meningitis, and cerebral infarction, with IgA nephropathy, tubulointerstitial nephritis, and glomerulonephritis being some of the renal manifestations [7].

The diverse and nonspecific clinical features of RP alongside its relative rarity frequently lead to a diagnostic delay [8] which is associated with the lack of ear, nose, or joint involvement but presents with early signs of intermittent arthritis or eye involvement in such cases [4]. RP patients may develop significant disabilities during the course of the disease such as hearing and visual impairments, and 30–50% of patients suffer pulmonary complications [9, 10].

The diagnosis of RP is mainly clinical using a classification criteria developed by McAdam and later expanded on by Damiani-Levine and Michet, which includes recurrent inflammation of both auricular, nasal, laryngeal and tracheal cartilages, noninflammatory arthritis, inflammation of ocular structures, and cochlear or vestibular damage [4]. The diagnosis can also be made from a positive biopsy of the ear, nasal, or respiratory cartilage [4, 7]. There are no laboratory investigations specific for the diagnosis of RP and laboratory findings may be suggestive of ongoing inflammation or organ damage revealing the presence of anemia, thrombocytosis, leukocytosis, or a derangement in renal function or urinalysis signifying renal impairment [7]. In RP, serological tests such as the rheumatoid factor, antinuclear antibodies (ANA), complement levels, extractable nuclear antibodies (ENA), and antiphospholipid antibodies are usually negative, but may be positive in the presence of other associated autoimmune conditions [11]. ANA is usually negative and can occur in low titers. The presence of significant titers of ANA in a patient with RP connotes a possibility of an associated autoimmune disorder [7]. Antineutrophil cytoplasmic antibodies (ANCA) may be present in about 25% of patients with RP which can be an incidental finding or the presence of an ANCA-associated vasculitis or herald the beginning of one [3].

The management of RP is mainly empirical as there are no evidence-based guidelines for treatment. The aim of therapy is to reduce inflammation through immunosuppression and prevent multiorgan damage to cartilaginous structures [7]. Mild disease is treated with low dose steroids, NSAIDs, colchicine, and dapsone while high dose steroids and conventional DMARDs such as methotrexate, azathioprine cyclophosphamide, or mycophenolate mofetil are employed in severe life-threatening flares [4]. Biologic therapy agents such as infliximab, etanercept, and adalimumab appear to show efficacy in the treatment of RP that is resistant to conventional DMARDs [12].

The disease entity continues to baffle the scientific community because of its rarity, unknown etiopathogenesis, clinical diversity, unpredictable progression, and uncodified treatment [1]. Coexistence of SCD and RP, an autoimmune disorder, poses a diagnostic and therapeutic challenge as patients with these conditions can present with symptoms that are similar in both conditions, for instance, fever, polyarthritis, and multiorgan involvement. Therefore, early diagnosis of RP is important to define the best treatment modality which may include targeted biological therapy [13].

RP in the setting of sickle cell disease can be difficult to diagnose as a result of the nonspecific nature of symptoms and presentation; therefore, a high index of suspicion is needed to make the diagnosis early and institute treatment to prevent the significant morbidity and mortality associated with the disease, especially in the pediatric group.

The purpose of this article is to highlight the fact that the disease can occur in the pediatric population and also in association with sickle cell disease. It also aims to reiterate the difficulty in managing such cases as there are no written protocols in the treatment of RP and that medications used in the treatment of RP can worsen SCD and its complications.

We describe a case of relapsing polychondritis in an 11-year-old girl with sickle cell disease (SF genotype) who suffered severe respiratory tract involvement, which posed a considerable management challenge. This case is of significant interest due to the rarity of the condition, the young age of our patient, and the association with sickle cell disease; previously unreported in the literature compared to the known coexistence with autoimmune conditions such as vasculitis, systemic lupus erythematosus, and rheumatoid arthritis, and the hurdles encountered during treatment.

## 2. Case Report

A 9-year-old young girl was referred to the rheumatology clinic from the ophthalmology unit as a case of systemic vasculitis with episcleritis. She presented with recurrent bilateral red painful eyes, a painful swollen left ear, and left knee pain in the preceding 10 months. A month prior to the presentation, she had developed an intermittent fever and reduced hearing. Three months prior to this, she had been admitted with painful swelling of the left knee and a fever, for which she was treated for septic arthritis of the left knee. Two years preceding the visit to the rheumatology clinic, she had also presented to the otorhinolaryngology unit on several occasions with complaints of hearing difficulty and was diagnosed with otitis externa. She has sickle cell disease-genotype SF and is a regular attendant at the pediatric sickle cell clinic. She did not suffer from an autoimmune condition, but had a first-degree cousin with systemic lupus erythematosus. She is the first of 2 children (her younger sibling is well) and has been unable to attend school due to arthralgia and difficulty hearing.

### 2.1. Clinical and Laboratory Findings

**Figure 1:**
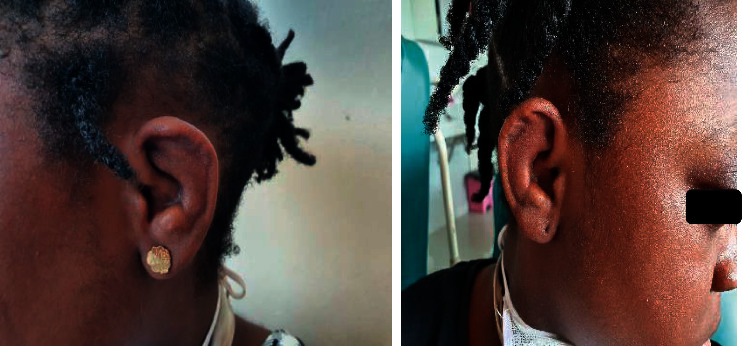
Swollen erythematous right and left ears with shriveled up outer ears resembling cauliflower ears.

**Figure 2:**
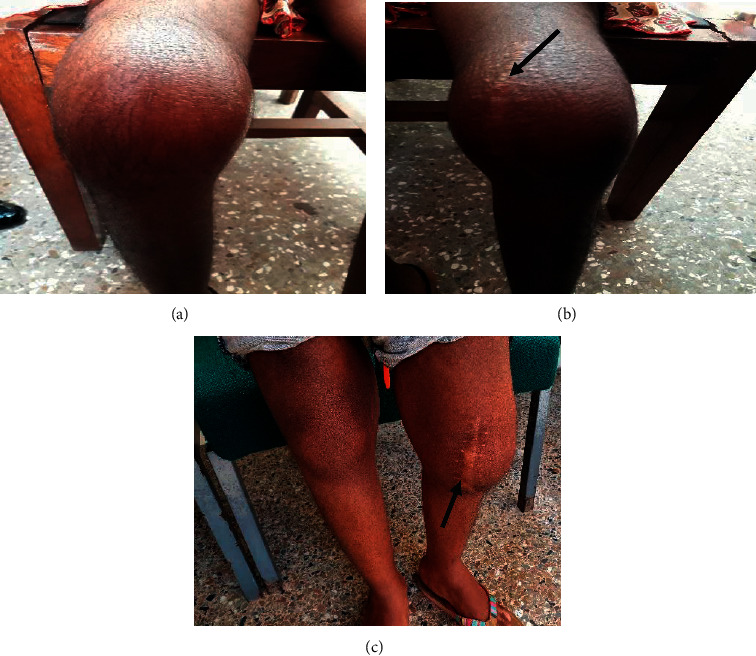
(a, b) Synovitis of the right and left knees when the patient was first seen at the outpatients' department. (c) A significant decrease in knee synovitis 2 months into treatment, with an old arthrotomy scar (arrowed).

On examination, there was mild pallor, axillary lymphadenopathy, and bilateral conjunctival injection with proptosis. The pinnae were swollen, erythematous, and tender, resembling “cauliflower ears” (Figure 1). The cardiorespiratory system examination was normal. There was acute synovitis of both knees with an old arthrotomy scar on the left (Figure 2).

Results from a previous synovial biopsy showed moderate, chronic inflammatory infiltrates composed of lymphocytes and plasma cells with stromal edema and congested vessels, but no malignant cells were seen, in keeping with chronic synovitis. The audiology assessment confirmed bilateral sensorineural hearing loss, worse in the left ear.

Laboratory investigations showed the following: Hb 10.7 g/dl (11–18 g/dl): microcytic, hypochromic pattern; WBC 15.7 × 10^9/L (2.5–8.5 × 10^9); and platelets 688 × 10^9/L (150–450 × 10^9). Erythrocyte sedimentation rate (ESR) and C reactive protein (CRP) were elevated at 96 mm/hr (0–15 mm/hr) and 143.7 mg/l (<5 mg/l), respectively. Hepatitis B, hepatitis C, and retroviral screens were negative. Hb electrophoresis–Hb A2–3%, Hb F–38.8%, Hb S–57.7%. The rheumatoid factor, ANA, and ENA panel were negative.

The initial diagnoses considered were juvenile idiopathic arthritis, septic arthritis of the left knee, systemic vasculitis, relapsing polychondritis (RP), and Cogan's syndrome, but the condition was subsequently confirmed to be RP with inflammatory arthritis based on the diagnostic criteria by McAdam et al. She initially fulfilled 4 out of the 6 features which are as follows: recurrent chondritis of both auricles, nonerosive inflammatory arthritis, inflammation of ocular structures, and bilateral sensorineural hearing loss, and later went on to develop an additional feature of chondritis of laryngeal and or tracheal cartilages as outlined below.

### 2.2. Clinical Course

She was admitted and started on intravenous (IV) clindamycin 180 mg 6 hourly and IV ciprofloxacin 200 mg 12 hourly for septic arthritis; oral ibuprofen 200 mg 12 hourly, oral paracetamol 250 mg 8 hourly, and prednisolone 15 mg daily to which she showed initial signs of response. The steroids and analgesia were started to help control the inflammation and pain associated with the disease. However, her disease flared up again with significant knee pain and swelling, a left conjunctival injection associated with pain, and high inflammatory markers. Prednisolone was increased from 15 mg to 20 mg daily, and oral methotrexate 7.5 mg weekly and folic acid 5 mg weekly were introduced. The inflammation in the eye persisted, though there was a reduction in inflammatory markers (ESR 70 mm/hr., CRP 111.7 mg/l) at subsequent reviews. The methotrexate dose was titrated up to 10 mg weekly, and the prednisolone dose was further increased to 30 mg daily. Two months after initiation of therapy, she had resolution of the auricular inflammation and a reduction in intermittent eye inflammation. Her knee swelling and tenderness had improved (Figure 2(c)), with a significant decline in her inflammatory markers (ESR-34 mm/hr, CRP 42.7 mg/l). The dose of methotrexate was increased further from 10 mg to 15 mg weekly. This was to provide steroid-sparing treatment, as she still had significant symptoms despite the higher doses of steroids used for control of inflammation. Despite the increase in the methotrexate dose, there was still significant disease activity after 2 months which signified some degree of corticosteroid dependence. The dose of prednisolone was tapered gradually at a dose of 5 mg weekly anytime there was a decrease in inflammatory markers, suggesting a reduction in inflammation.

Due to failure to respond adequately to methotrexate, as evidenced by the frequency of disease flares and worsening of her eye symptoms, she also developed cataracts due to the high doses of steroids used. This led to the introduction of infliximab 10 months into treatment. She received the loading course of 100 mg of intravenous infliximab at 0, 2, and 6 weeks, but was unable to continue with the maintenance therapy of 8-weekly infusions due to financial constraints. Despite the short course of infliximab, clinical benefits such as reduction in arthritis and eye inflammation were noted. Six weeks after her last infliximab infusion, she developed difficulty breathing.

This progressed, leading to her presenting in the emergency room with signs of respiratory distress. On examination, she had audible inspiratory stridor, tachypnea, inspiratory rhonchi, and a slight reduction in breath sounds bilaterally, though vesicular in nature.

**Figure 3:**
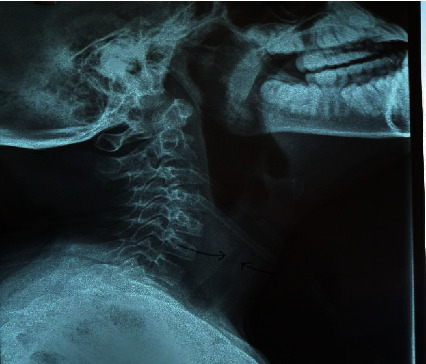
Lateral cervical X-ray showed partial narrowing in the region of the cricoid cartilage (arrowed).

Lateral cervical X-ray showed partial narrowing in the region of the cricoid cartilage (Figure 3). A diagnosis of upper airway obstruction (subglottic) secondary to perichondritis of the cricoid cartilage with respiratory fatigue was made upon review by the otorhinolaryngologist and pulmonologist. Treatment ensued with intravenous dexamethasone 2 mg 8 hourly, nebulized Pulmicort 500 mcg 12 hourly, and salbutamol 2.5 mg 4 hourly, and she was transferred to the intensive care unit. An emergency tracheostomy to relieve the subglottic stenosis was performed. She recovered well and was discharged on day 4 of admission.

Her medications were reviewed, taking into account the financial challenges to include oral colchicine 500 mcg twice daily, oral methotrexate 15 mg weekly and oral prednisolone 40 mg daily which was to be tapered gradually. Knee synovitis persisted, requiring intra-articular injections of 80 mg of methylprednisolone acetate into each joint. Tracheostomy care was carried out regularly at the otorhinolaryngology department, and close vigilance for infection was maintained.

**Figure 4:**
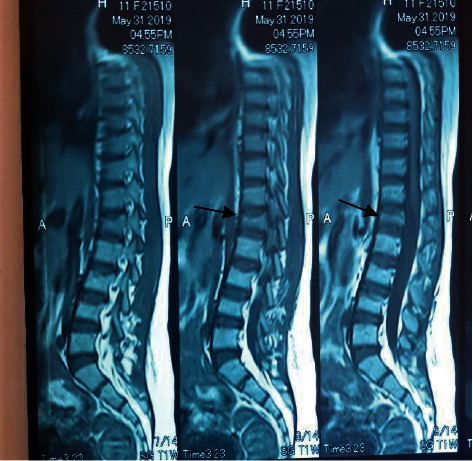
Compression fracture at the L1 vertebra (arrowed), with similar but milder changes in the T11, L3, and L4 vertebrae suggestive of a bone softening process due to glucocorticoid-induced osteoporosis and underlying hemoglobinopathy a year and a half into the treatment of her condition.

A year and a half from the start of her treatment, she developed mid- to low-back pain. An X-ray of the lumbosacral spine showed a vertebral collapse at the L1 vertebra. A compression fracture was confirmed on MRI, with similar but less pronounced changes seen in T11, L3, and L4 vertebrae, suggestive of a bone softening process in keeping with glucocorticoid-induced osteoporosis and underlying hemoglobinopathy (Figure 4).

Steroid dose reduction continued and bisphosphonate therapy with alendronate 35 mg weekly was introduced. Oral dapsone 25 mg daily was added to her treatment for disease control due to its immunosuppressive and anti-inflammatory properties, but she continues to have remitting and relapsing disease.

## 3. Discussion

Relapsing polychondritis is an uncommon disorder of unknown cause characterized by inflammation of cartilage predominantly affecting the ears, nose, and laryngotracheobronchial tree which is among the most serious though less common manifestations of RP [14]; all of which manifested in the 11-year-old child as above mentioned. Other features she had were scleritis, sensorineural hearing loss, and arthritis.

RP can cause cardiac abnormalities such as valvular regurgitation, aneurysms, pericarditis, myocarditis, coronary vasculitis, and conduction abnormalities. Skin lesions such as purpura, erythema nodosum or multiforme, urticaria, livedo reticularis, and panniculitis can also occur in patients with RP with renal disease manifesting in the form of mesangial expansion or segmental necrotising glomerulonephritis [15].

The peak age of incidence is between the fourth and the fifth decade of life, but relapsing polychondritis may affect children and the elderly alike [8] and occurs with similar frequency in both genders although a slight female predominance has been cited. RP has documented cases in all ethnic groups, but most cases appear to be of Caucasian origin with no apparent familial tendency [5].

Pediatric RP represents less than 5% of the cases reported, with the age at onset varying from 1.7 months to 17 years. The clinical presentation is similar to that of adults, but there is a higher rate of severe airway disease in the pediatric population, and it commonly affects females [14]. About 30% of patients with RP will also have another rheumatologic disorder, the most frequent being systemic vasculitis, followed by rheumatoid arthritis and systemic lupus erythematosus. Children with RP frequently have a family history of autoimmunity and usually do not have associated autoimmune diseases [4], as is seen in our patient.

Nonrheumatic conditions such as inflammatory bowel disease, myelodysplastic syndrome, and Grave's disease have been associated with RP, and these conditions may predate the appearance of RP by months or years or can occur concurrently [1]. There are currently no reports of sickle cell disease associated with RP as our patient had, and whether it is part of the group of nonrheumatic diseases is yet to be established.

Vaso-occlusive crises (VOC) of SCD can mimic RP arthritis and the two can be particularly difficult to distinguish clinically since both SCD and RP have involvement of the small and large joints; however, patients with RP usually have asymmetric nonerosive intermittent arthritis of these joints with axial sparing [15]. SCD arthritis is usually symmetric, with over 60% of cases having a preference for large joints and the lower extremity bones [16].

With the coexistence of these two disease entities in the setting of developing arthritis, determining whether it is RP arthritis or a VOC with superimposed inflammation resulting from local phagocytosis of sickled erythrocytes can be challenging. This phenomenon may drive the development of inflammatory arthritis in that synovial ischemia can lead to the release of synovial antigens into circulation and subsequently lead to the activation of autoantibodies against the synovium of the SCD patient, leading to inflammatory arthritis. Recurrent VOC in SCD can lead to a continuous inflammatory response that shares many characteristics and cytokines with arthritis of inflammatory origin, an example being interleukin 1 and 6, which are common to both of them [17].

The diagnosis of RP is a real challenge for physicians because of its diverse nature and insidious onset. The multiple clinical presentations and episodic nature of RP cause a significant delay in diagnosis, ranging from 1.9 to 10 years, with an average of five physicians being consulted before diagnosis, particularly in the pediatric population [8]. This delay is even more significant in patients with SCD, as they share some nonspecific symptoms such as arthralgia, fever, and multiorgan involvement. In addition, the poor knowledge of this condition by pediatricians may account for a mean delay of five years [14]. It is therefore recommended to have a sturdy respiratory and cardiovascular surveillance plan in all patients with early or associated pulmonary features to help delay disease progression [15] and reduce morbidity and mortality.

The diagnosis is certain when three or more of the clinical features listed in Table 1 are present along with a positive biopsy from the ear, nasal, or respiratory cartilage. A positive biopsy is only necessary when the clinical features are not typical.

The diagnosis can also be made when one or more of the above features and a positive biopsy are present, or when two or more separate sites of cartilage inflammation are present and respond to steroids or dapsone; or when three or more of the above features are present according to Damiani and Levine [18].

From Table 1, recurrent chondritis of both auricles is the most common feature of RP, being present in up to 90% of patients during the course of the disease, with nonerosive inflammatory arthritis being the second most common feature. Clinicians can therefore look out for chondritis of the ear in the early phase of the condition with repeated flares leading to severe destruction of the cartilage and subsequent loss of normal morphology of the pinna. This deformity resembles the “cauliflower ear” of professional boxers in a small percentage of patients [7].

The presence of inflammation in 2 or 3 cartilages of either the auricular, nasal, or laryngotracheal structures confirms the diagnosis of RP, or the confirmation of inflammation in one of the above listed cartilages plus 2 other minor criteria, namely, hearing loss, ocular inflammation, vestibular dysfunction, or seronegative arthritis, clinches the diagnosis of RP [19]. These criteria were used to finalize the diagnosis of RP in the case presented, as she had 5 out of the 6 diagnostic criteria according to McAdam.

This young patient with RP exhibited significant disease activity and severity with complex presentations which has led to management challenges with no breakthrough in therapies administered. The management of RP is mainly empirical due to the lack of understanding of the etiopathogenesis, absence of clinical trials or validated treatment recommendations, and the rarity of the disease entity [1]. First-line therapy involves the use of corticosteroids, dapsone, colchicine, and nonsteroidal anti-inflammatory drugs. Steroid-sparing medications such as methotrexate, cyclophosphamide, azathioprine, chlorambucil, mycophenolate mofetil, minocycline, and leflunomide may be used as second-line therapy and have been shown to be effective in some patients with steroid-resistant or steroid-dependent and/or life-threatening disease. They are also used to decrease the adverse effects of excessive steroid therapy [1, 15]. From the case discussion, our patient was started on most of the medications aforementioned with methotrexate being the only steroid-sparing medication used as some of the others were either expensive (mycophenolate) or not available in the country (leflunomide) with no notable improvement in disease condition with significant steroid dependence occurring in an attempt to control the disease. The refractory nature of the disease had resulted in persistently high doses of prednisolone being used, leading to complications of steroid-induced cataracts, osteoporosis, and vertebral fracture developing. Patients with SCD have decreased bone density due to erythroid hyperplasia from increased hematopoietic activity in bone resorption which invariably leads to generalized osteoporosis and cortical thinning [16]. This was further worsened with resultant vertebral fracture in our patient who needed high dose steroids to control her disease state. Biologics seem to be the new artillery in the war against autoimmune rheumatic disorders, like RP [7, 15] with case reports supporting its use in treating young patients with episcleritis and tracheal chondritis [20].

The most common biologic prescribed in the management of RP is infliximab, with adalimumab and tocilizumab being the next most prescribed in second and third place, respectively. Other biologics that have been used include rituximab, etanercept, and anakinra [7, 12]. The effectiveness of biologic therapy in RP is mixed, spanning from a good response to a complete lack of efficacy [12]; some patients with RP may have resolution of symptoms with biologics, while others will not. Infliximab, which has been used as a third-line agent [15], could not be pursued for this patient due to significant financial constraints, even though it showed a significant response. Colchicine and dapsone were started as a result of their reported efficacy in RP management [1] and were the next cheapest and readily available agents in our setting as the patient could not afford continuation of biologic therapy with Infliximab. In resource-limited areas such as in our setting, a few conventional and biologic DMARDs are available; namely, methotrexate, azathioprine, mycophenolate mofetil, and cyclophosphamide, with infliximab, rituximab, and only recently, tocilizumab, as the biologic artillery.

## 4. Conclusion

RP is an infrequent multisystemic, potentially lethal autoimmune condition of unknown etiology, affecting cartilaginous and proteoglycan-rich tissues in an episodic pattern with treatment being empiric. Though rare, especially in the young, we report on an unusually young patient who had sickle cell disease as a comorbidity and an aggressive clinical course of RP, with complications due to both disease and treatment. Diagnostic delays increase morbidity and mortality associated with both diseases; hence, early recognition and diagnosis are paramount to enable prompt treatment for improved clinical outcomes. The concomitant use of steroids in the management of RP though beneficial worsened severe SCD associated complications which justified the need for other therapies with cost being a significant limitation.

Further studies are needed to determine the prevalence of RP in patients with SCD and any pathophysiological links and also to define therapeutic options or modalities in managing patients with both conditions.

## Figures and Tables

**Table 1:** Diagnostic criteria for RP according to McAdam et al. [9], and the observed frequencies of clinical presentation in patients, during the course of the disease [7].

1. Recurrent chondritis of both auricles (90%)
2. Chondritis of nasal cartilage (53%)
3. Nonerosive inflammatory arthritis (50–85%)
4. Chondritis of laryngeal and or tracheal cartilages (50%)
5. Inflammation of ocular structures, including conjunctivitis, keratitis, scleritis/episcleritis, and/or uveitis (50–60%)
6. Cochlear and or vestibular (6%) damage manifested by neurosensory hearing loss (46%), tinnitus, and/or vertigo
